# THE BURDEN OF COGNITIVE IMPAIRMENT

**DOI:** 10.1093/geroni/igad104.1729

**Published:** 2023-12-21

**Authors:** Ritika Chaturvedi, Tadeja Gracner, Hanke Heun-Johnson, Bryan Tysinger, Dana Goldman

**Affiliations:** USC, Los Angeles, California, United States; RAND Corporation, Santa Monica, California, United States; University of Southern California, Los Angeles, California, United States; University of Southern California, Los Angeles, California, United States; University of Southern California, Los Angeles, California, United States

## Abstract

Over 10% of American seniors (age≥65) experience cognitive impairment (CI) related to Alzheimer’s disease and related dementias (ADRD). An additional 15-22% experience mild cognitive impartment (MCI), which often precedes ADRD and occurs earlier in life. However, CI burden estimates inclusive of MCI and early CI onset (age< 65) are limited. Using microsimulation, we estimated the burden of CI (ADRD and/or MCI) over remaining life years above 50 overall and by disease progression, age of onset, and sociodemographic subgroups for a nationally representative sample of Americans (age≥50) with cognitive status information in the Health and Retirement Study (2000-2016). The per capita lifetime CI burden was $124K (95%CI:$101K-$148K) due to $162K (95%CI:$139K-$185K) and $6.40K (95%CI:$5.03K-$7.77K) in lost Quality Adjusted Life Years (QALYs) and earnings, respectively, and $4.07K (95%CI:$1.82K-$6.33K) in informal care costs, offset by $48.6K (95%CI: $42.4K-$54.8K) in lower medical expenditures. The aggregate CI burden was $627B (95%CI: $511B–$743B): 59% due to ADRD and 41% to MCI. Individuals with MCI without progression to ADRD lost healthy life years valued at $119K (95%CI:$99K-$139K). Those with early CI onset were more likely to be from disadvantaged populations and carried the largest per capita burden [$376K (95% CI: $315K-$436K)] cumulating to 60% on aggregate, nearly half of which experienced during MCI. Individuals with lower education, progression to ADRD, or racial/ethnic minorities also carried disproportionate burden. MCI represents 41% of the overall CI burden and disproportionately affects individuals with early CI onset. Characterizing CI-related health and economic disparities at milder stages and younger ages should be prioritized.

